# Antenatal visit model in low-risk pregnancy during pandemic COVID-19: A call for adjustments

**DOI:** 10.18332/ejm/121097

**Published:** 2020-05-07

**Authors:** Danai Sklaveniti

**Affiliations:** 1 Midwifery Department, University of West Attica, Athens, Greece

**Keywords:** COVID-19, pregnancy, antenatal, telemedicine, teleconsultation

**Dear Editor,**

Pregnancy is a natural situation in every woman’s life. Midwifery is linked to more effective utility resources and improved health outcomes when it is conducted by certified, educated and trained midwives that comply to integrated and preventive antenatal care^1^.

Low-risk pregnancy antenatal care is usually followed by ten appointments for nulliparous women and seven appointments for parous^2^. Higher number of appointments do not correlate with better maternal and fetal outcomes^3^. Except for fetal ultrasounds, which are carried out by trained doctors, routine visits should be provided by a midwife in-person or through telephone/virtual consultations^4^ during pregnancy. On account of the COVID-19 pandemic, it is an urgent need to reduce antenatal appointments to a minimum of six but no fewer than that, although evidence indicates that five or fewer visits are connected with increased risk of perinatal mortality in countries of low or middle income^4^. According to the United Nations Population Fund, by reducing the number of facility visits, the risk of exposure and virus transmission is also minimized. Midwives should continue to provide maternity care within the community to avoid accumulation of adverse maternal and newborn outcomes and also use remote means such as phone and mobile communication applications when physical presence, examination or tests are not necessary^5^.

We knew little up until now about the impact of COVID-19 in pregnant women and the fetuses. However, awareness among health providers should be raised, since the pandemic outbreak will reach its end at some point, but meanwhile there will be many aspects in need of tending to, such as maternal stress, depression and domestic violence because of socioeconomic changes during the pandemic^5^. Maternity care should remain primary in the context of the maternal/child continuum healthcare model^6^. ‘It is important that care is available to ensure continuation of support for women with multiple complex needs. Women living with adversity including poverty, homelessness, substance misuse, being an asylum seeker, experiencing domestic abuse and mental health problems will continue to require timely expert support’, as recommended by the Royal College of Obstetricians and Gynaecologists^7^. The International Confederation of Midwives has also expressed concern regarding the necessity for sustaining the rights of all pregnant women and their newborns by following evidence-based practices and protocols^8^ in general, especially in these challenging times to avoid malpractice and unnecessary medicalization.

## CONFLICTS OF INTEREST

The author has completed and submitted the ICMJE Form for Disclosure of Potential Conflicts of Interest and none was reported.
